# Identification of multiple complications as independent risk factors associated with 1-, 3-, and 5-year mortality in hepatitis B-associated cirrhosis patients

**DOI:** 10.1186/s12879-025-10566-6

**Published:** 2025-02-01

**Authors:** Duo Shen, Ling Sha, Ling Yang, Xuefeng Gu

**Affiliations:** 1 Department of Gastroenterology, The Second People’s Hospital of Changzhou, the Third Affiliated Hospital of Nanjing Medical University, Changzhou, Jiangsu China; 2 Department of Neurology, Nanjing Drum Tower Hospital, Affiliated to Nanjing University Medical School, Nanjing, Jiangsu China; 3 Department of Central Laboratory, Jurong Hospital Affiliated to Jiangsu University, 66 Ersheng Road, Jurong, Zhenjiang, Jiangsu 212400 China; 4 Department of Infectious Diseases, Jurong Hospital Affiliated to Jiangsu University, 66 Ersheng Road, Jurong, Zhenjiang, Jiangsu 212400 China

**Keywords:** Hepatitis B, Cirrhosis, Complications, Machine learning, GBDT, Survival analysis

## Abstract

**Background:**

Hepatitis B-associated cirrhosis (HBC) is associated with severe complications and adverse clinical outcomes. This study aimed to develop and validate a predictive model for the occurrence of multiple complications (three or more) in patients with HBC and to explore the effects of multiple complications on HBC prognosis.

**Methods:**

In this retrospective cohort study, data from 121 HBC patients treated at Nanjing Second Hospital from February 2009 to November 2019 were analysed. The maximum follow-up period was 10.75 years, with a median of 5.75 years. Eight machine learning techniques were employed to construct predictive models, including C5.0, linear discriminant analysis (LDA), least absolute shrinkage and selection operator (LASSO), k-nearest neighbour (KNN), gradient boosting decision tree (GBDT), support vector machine (SVM), generalised linear model (GLM) and naive Bayes (NB), utilising variables such as medical history, demographics, clinical signs, and laboratory test results. Model performance was evaluated via receiver operating characteristic (ROC) curve analysis, residual analysis, calibration curve analysis, and decision curve analysis (DCA). The influence of multiple complications on HBC survival time was assessed via Kaplan‒Meier curve analysis. Furthermore, LASSO and univariable and multivariable Cox regression analyses were conducted to identify independent prognostic factors for overall survival (OS) in patients with HBC, followed by ROC, C-index, calibration curve, and DCA curve analyses of the constructed prognostic nomogram model. This study utilized bootstrap resampling for internal validation and employed the Medical Information Mart for Intensive Care IV (MIMIC-IV) database for external validation.

**Results:**

The GBDT model exhibited the highest area under the curve (AUC) and emerged as the optimal model for predicting the occurrence of multiple complications. The key predictive factors included posthospitalisation fever (PHF), body mass index (BMI), retinol binding protein (RBP), total bilirubin (TB) levels, and eosinophils (EOS). Kaplan–Meier analysis revealed that patients with multiple complications had significantly worse OS than those with fewer complications. Additionally, multivariable Cox regression analysis, informed by least absolute shrinkage and LASSO selection, identified hepatocellular carcinoma (HCC), multiple complications, and lactate dehydrogenase (LDH) levels as independent prognostic factors for OS. The prognostic model demonstrated 1-year, 3-year, and 5-year OS ROC AUCs of 0.802, 0.793, and 0.817, respectively. For the internal validation cohort, the corresponding AUC values were 0.797, 0.832, and 0.835. In contrast, the external validation cohort yielded a 1-year ROC AUC of 0.707. Calibration curves indicated good consistency of the model, and DCA demonstrated the model’s clinical utility, showing high net benefits within certain threshold ranges. Compared with the univariable models, the multivariable ROC curves indicated higher AUC values for this prognostic model, and the model also possessed the best c-index.

**Conclusion:**

The GBDT prediction model provides a reliable tool for the early identification of high-risk HBC patients prone to developing multiple complications. The concurrent occurrence of multiple complications is an independent prognostic factor for OS in patients with HBC. The constructed prognostic model demonstrated remarkable predictive performance and clinical applicability, indicating its crucial role in enhancing patient outcomes through timely and targeted interventions.

**Supplementary Information:**

The online version contains supplementary material available at 10.1186/s12879-025-10566-6.

## Introduction

Infection with hepatitis B virus (HBV) remains a formidable global health challenge, particularly given its propensity to progress to cirrhosis and associated complications. In 2019, HBV-related cirrhosis resulted in approximately 331,000 fatalities worldwide [1]. Globally, 42% of cirrhosis patients suffer from HBV infection [2], with prevalence rates varying significantly across regions, including 8-61% in Africa and Asia compared with 3-14% in Europe, the Americas, and Oceania [3]. Notably, in China, 68% of cirrhosis cases are attributed to HBV infection [4]. Cirrhosis, specifically hepatitis B-associated cirrhosis (HBC), leads to significant morbidity and mortality largely due to its complications. The latest Global Burden of Disease study in 2019 reported an 8.1% increase in global cirrhosis deaths since 2017, totalling 1.43 million [5]. The epidemiology of major cirrhosis complications includes ascites, variceal haemorrhage, hepatic encephalopathy (HE), renal diseases, and infections [3]. Despite advancements in HBV treatment, managing cirrhosis and its sequelae remains a substantial challenge for health systems, highlighting the urgent need for effective predictive strategies.

Recent research has emphasised the development of predictive models capable of effectively identifying the onset of severe complications and assessing survival in cirrhosis patients [6–10]. An array of such models has emerged, fostering early interventions that could improve outcomes and resource allocation for patients with cirrhosis. However, despite advancements in predictive analytics, few models have been effectively constructed and validated for the prediction of multiple complications (three or more) in HBC patients.

Machine learning (ML) algorithms, a subset of artificial intelligence, have demonstrated significant advantages in predicting or classifying outcomes on the basis of input data [11]. Predictive models based on ML algorithms have been developed for various aspects of HBC patient treatment [6–10]. In constructing predictive models, ML excels by efficiently analysing and interpreting large and complex datasets to identify prognostic factors and predict disease progression trajectories [12–14]. However, to date, no study has applied these methods to predict the occurrence of multiple complications in HBC patients. Our research aims to analyse admission data, screen for early risk factors associated with multiple complications in HBC patients, and construct predictive models using ML algorithms to identify patients at high risk of severe complications at the early stages of hospital admission. However, research addressing the long-term prognostic implications of multiple complications in HBC patients is scarce. Our work extends beyond merely constructing a predictive model for the occurrence of multiple complications in HBC using machine learning. This study also evaluated the impact of these complications on the overall survival (OS) of HBC patients with long-term prognostic follow-up. Importantly, these complications have been integrated as risk factors in the development of a nomogram model for predicting OS in HBC patients, thereby enhancing its utility in clinical settings.

This study leverages data from Nanjing Second Hospital (February 2009 - November 2019) to develop a ML prognostic model for predicting multiple complications in HBC patients. Model performance is validated through receiver operating characteristic (ROC) curves, residual analysis, decision curve analysis (DCA), and calibration curves. Additionally, the study investigates the impact of multiple complications on OS in HBC patients, using univariate Cox, least absolute shrinkage and selection operator (LASSO), and multivariate Cox regression to identify independent prognostic indicators. A prognostic nomogram is also constructed to enhance individualised patient management. Overall, the study not only provides a reliable predictive model for HBC complications but also highlights their significance in patient survival, contributing to improved prognostication and personalised care strategies.

## Materials and methods

### Data collection

This retrospective cohort study involved 121 HBC patients from Nanjing Second Hospital (February 2009 - November 2019), with a follow-up lasting up to 10.75 years (median 5.75 years). Data with over 90% missing values were excluded, and missing data were imputed using R’s “rpart” package.

### Variables and outcomes

We selected 57 independent variables based on patient history, demographics, clinical signs, and laboratory results. The dependent variable for the predictive model was the occurrence of multiple complications, including ascites, variceal bleeding, hepatic encephalopathy, renal diseases, and infections. Survival time and status were the outcome variables for the prognostic model of HBC patients. Ascites diagnosis followed European Association for the Study of the Liver (EASL) criteria [15], classifying them into three grades based on severity and excluding non-cirrhotic causes. Hepatic encephalopathy was classified according to West-Haven criteria, ranging from stage 0 (no symptoms) to stage 4 (coma), with a focus on excluding other causes of altered mental state [16]. Hepatorenal syndrome was diagnosed per EASL standards [15]: presence of cirrhosis, a significant increase in serum creatinine, no kidney function improvement through volume expansion, and absence of nephrotoxic drug use. Concomitant gastrointestinal bleeding was identified via cirrhosis diagnosis and variceal bleeding signs during endoscopy [17]. Spontaneous bacterial peritonitis (SBP) was diagnosed with a positive ascitic fluid culture or a neutrophil count ≥ 250 cells/mm³, excluding other infections. Infectious complications included pulmonary and urinary tract infections, severe skin infections, bacteraemia, and sepsis [15]. Survival time was defined from HBC diagnosis to death or study completion. Internal validation was conducted using R’s “boot” package, a recognised method for rigorous internal validation of predictive models [18–21].

The training cohort (Table 1) consisted of 121 participants, with 92 males (76.0%) and 29 females (24.0%), aged 7 to 77 years (mean age 53.69 ± 12.13 years). A family history of hepatitis B was noted in 27 patients (22.3%), and lifestyle factors included alcohol consumption in 11 (9.1%) and smoking in 9 (7.4%). HCC was diagnosed in 56 patients (46.3%), with hypertension and diabetes affecting 19 (15.7%) and 20 (16.5%), respectively. Common clinical signs included palmar erythema and Spider Nevi, observed in 93 (76.9%) and 37 (30.6%) patients. Post-hospitalisation fever and vomiting were reported in 52 (43.0%) and 47 (38.8%) patients, respectively. Most patients (90.9%) received nucleos(t)ide analogue antiviral therapy, and 63.6% were HBV DNA positive, with a median body mass index (BMI) of 23.4. The internal validation cohort also included 92 males (76.0%) and 29 females (24.0%), aged 17 to 77 years (mean age 55.68 ± 11.22 years). Family history, smoking, and alcohol consumption were reported in 18 (14.9%), 12 (9.9%), and 6 (5.0%) patients. HCC was present in 60 (49.6%), hypertension in 17 (14.0%), and diabetes in 27 (22.3%). Palmar erythema and Spider Nevi were noted in 96 (79.3%) and 40 (33.1%) patients, with post-hospitalisation fever in 53 (43.8%). Most (89.3%) received antiviral therapy, 64.5% tested HBV DNA positive, and the median BMI was 23.4. Both cohorts were comparable, with 46.3% and 47.1% experiencing three or more complications, respectively, with no significant differences in baseline complications (*p* < 0.05) (Supplementary Table 1).

**Table 1:** Demographic and clinical characteristics of hepatitis B-associated cirrhosis patients

Variables	Value
N	121
Gender	
Woman	29 (24.0%)
Male	92 (76.0%)
HBVFH	
No	94 (77.7%)
Yes	27 (22.3%)
EtOH	
No	110 (90.9%)
Yes	11 (9.1%)
SH	
No	112 (92.6%)
Yes	9 (7.4%)
HCC	
No	65 (53.7%)
Yes	56 (46.3%)
HTN	
No	102 (84.3%)
Yes	19 (15.7%)
DM	
No	101 (83.5%)
Yes	20 (16.5%)
PE	
No	28 (23.1%)
Yes	93 (76.9%)
SV	
No	84 (69.4%)
Yes	37 (30.6%)
PHF	
No	69 (57.0%)
Yes	52 (43.0%)
vomiting	
No	74 (61.2%)
Yes	47 (38.8%)
AVT	
No	11 (9.1%)
Yes	110 (90.9%)
HBVDNA	
negative	44 (36.4%)
positive	77 (63.6%)
Age (years, median IQR)	55.0 (45.0, 62.0)
BMI (median IQR)	23.4 (21.1, 25.4)
WBC (10^9/L, median IQR)	4.3 (2.9, 6.4)
NEU (10^9/L, median IQR)	2.8 (1.6, 4.3)
NLR (median IQR)	2.8 (1.8, 4.6)
HB (g/L, median IQR)	121.0 (107.0, 134.0)
PLT (10^9/L, median IQR)	67.0 (46.0, 100.0)
LYM (10^9/L, median IQR)	1.0 (0.7, 1.3)
PLR (median IQR)	71.8 (46.6, 101.4)
RBC (10^12/L, median IQR)	3.7 (3.2, 4.2)
HCT (%,median IQR)	35.4 (31.0, 39.3)
MONO (10^9/L, median IQR)	0.3 (0.2, 0.5)
EOS (10^9/L, median IQR)	0.1 (0.0, 0.1)
TB (µmol/L, median IQR)	24.0 (16.3, 37.3)
DB (µmol/L, median IQR)	10.5 (7.1, 17.5)
ALT (U/L, median IQR)	40.6 (26.2, 66.1)
AST (U/L, median IQR)	47.8 (30.4, 84.1)
ALB (g/L, median IQR)	32.8 (28.9, 37.6)
GLO (g/L, median IQR)	31.5 (26.5, 35.9)
GGT (U/L, median IQR)	67.3 (31.7, 150.3)
CHE (U/L, median IQR)	3507.0 (2242.0, 4896.0)
ALP (U/L, median IQR)	99.9 (67.1, 140.0)
LDH (U/L, median IQR)	210.0 (163.0, 251.0)
TBA (µmol/L, median IQR)	22.7 (9.2, 53.7)
Lactate (mmol/L, median IQR)	2.7 (2.2, 3.0)
RBP (mg/L, median IQR)	19.5 (13.3, 28.5)
AFU (U/L, median IQR)	19.0 (15.0, 25.0)
MAO (U/L, median IQR)	4.9 (3.5, 6.5)
ADA (U/L, median IQR)	24.1 (17.8, 31.8)
B2M (mg/ml, median IQR)	2.9 (2.2, 4.0)
Serum potassium (mmol/L, median IQR)	3.8 (3.6, 4.2)
Serum sodium (mmol/L, median IQR)	140.1 (137.3, 142.5)
Serum calcium (mmol/L, median IQR)	2.0 (1.9, 2.2)
Serum ferritin (umol/L, median IQR)	19.1 (9.9, 28.7)
Serum phosphate (mmol/L, median IQR)	1.0 (0.9, 1.1)
BUN (mmol/L, median IQR)	5.6 (4.7, 7.4)
Cr (µmol/L, median IQR)	69.9 (58.0, 79.0)
PT (second, median IQR)	14.8 (13.7, 16.8)
APTT (second, median IQR)	36.5 (30.9, 42.7)
TT (second, median IQR)	21.1 (19.7, 23.2)
Fib (g/L, median IQR)	1.7 (1.4, 2.3)
AT3 (mg/dl, median IQR)	73.2 (53.4, 85.3)
PTA (%,median IQR)	59.4 (50.8, 69.6)
AFP (ng/ml, median IQR)	20.7 (4.3, 239.7)

Note. ADA, adenosine deaminase; AFP, alpha-fetoprotein; AFU, alpha-l-fucosidase; ALB, albumin; ALP, alkaline phosphatase; ALT, alanine aminotransferase; APTT, activated partial prothrombin time; AST, aspartate aminotransferase; AT3, antithrombin III; AVT, Antiviral Therapy; B2M, Beta-2-microglobulin; BMI, Body Mass Index; BUN, blood urea nitrogen; CHE, cholinesterase; Cr, creatinine; DB, direct bilirubin; DM, Diabetes Mellitus; EOS, eosinophil; EtOH, Ethanol/Alcohol History; Fib, fibrinogen; GGT, γ-glutamyl transpeptadase; GLO, globulin; HB, hemoglobin; HBVFH, Family History of Hepatitis B Virus; HCC, Hepatocellular Carcinoma; HCT, hematocrit; HTN, Hypertension; IQR, interquartile range; LA, lactic acid; LDH, lactic dehydrogenase; LYM, lymphocyte; MAO, monoamine oxidase; MONO, monocyte; NEU, neutrophil; NLR, neutrophil to lymphocyte ratio; PE, Palmar Erythema; PHF, Post-hospitalisation fever; PLR, platelet to lymphocyte ratio; PLT, platelet; PT, Prothrombin time; PTA, prothrombin time activity; RBC, red blood cell; RBP, retinol binding protein; SH, Smoking History; SV, Spider Nevi; TB, total bilirubin; TBA, total bile acid; TBIL, total bilirubin; TT, thrombin time; WBC, white blood cell

### Construction and validation of the multiple complications predictive model

We utilised a range of machine learning algorithms—C5.0, linear discriminant analysis (LDA), LASSO, k-nearest neighbour (KNN), gradient boosting decision tree (GBDT), support vector machine (SVM), generalised linear model (GLM) and naive Bayes (NB)—to construct predictive models for identifying multiple complications in hepatitis B-related cirrhosis (HBC) patients. Each algorithm was selected based on its suitability for our dataset. The C5.0 algorithm builds decision trees by optimising information gain and is implemented using the “Caret” package [22, 23]. KNN predicts outcomes based on neighbouring samples, using the “kknn” package [24]. SVM establishes decision boundaries using the “max-margin” principle and the “kernlab” package, suitable for both linear and nonlinear classifications [25]. GLM links response variables and predictors through a link function, developed using the “Caret” package [26]. LASSO, ideal for variable selection in collinear data, is constructed via the “glmnet” package [27]. LDA identifies feature combinations for classification using the “e1071” package [28]. GBDT, a leading algorithm for capturing complex relationships via iterative decision trees, is implemented with the ‘xgboost’ package [29, 30]. NB utilises Bayes’ theorem for predictions, employing the “e1071” package [31]. Model performance was assessed through ROC analysis and residual assessments. Among these, the GBDT was selected as the final model due to its outstanding performance, demonstrating the highest area under the ROC curve (AUC of 0.928), minimal residual errors, and robustness in capturing nonlinear relationships. Unlike linear models such as GLM and LDA, GBDT manages intricate data complexities effectively, automatically learns feature interactions, and offers feature importance analyses for interpretability. High-dimensional data processing benefits from GBDT’s ability to select significant features and the flexibility provided by fine-tunable hyperparameters, setting it apart from models like C5.0 and LASSO that have fewer hyperparameter options. The identified optimal risk variables from the GBDT model were used to construct a nomogram, aiding in the estimation of multiple severe complication risks and guiding clinical decisions. The model’s consistency with observed probabilities and clinical applicability were further validated through concordance curves and DCA.

### HBC patient mortality risk assessment

The relationships between prognosis and multiple complications in HBC patients were examined using Kaplan-Meier survival analysis for estimating survival probabilities. We analysed survival differences among patients with varying complication counts using Kaplan-Meier curves. Univariate Cox regression was conducted on 58 variables to identify prognostic factors (*p* < 0.05). To prevent overfitting, LASSO regression, implemented via the R package glmnet with 3-fold cross-validation over 1000 iterations, was employed to select significant variables by penalising and shrinking some coefficients to zero. A prognostic risk model was constructed using multivariable Cox regression with the Akaike Information Criterion (AIC) to identify independent prognostic factors, adjusting for confounding factors. This multi-step process, involving univariate Cox, LASSO, and multivariable Cox regression, resulted in the identification of independent prognostic factors for overall survival and the construction of a nomogram model. Model assessment was performed using calibration curves, time-dependent ROC, multi-parameter timeROC, and DCA. An internal validation cohort of 121 patients, created via bootstrap resampling, revealed no significant differences compared to the original training cohort, ensuring comparability (*p* > 0.05) (Supplementary Table 2).

### External validation using medical information mart for intensive care IV (MIMIC-IV) data

In this retrospective external validation cohort study, we utilized the MIMIC-IV version 3.1 (released October 2024), a comprehensive publicly accessible dataset that provides post-discharge mortality data up to one year. This repository includes clinical information for 364,627 patients and 546,028 hospital admissions recorded at the Beth Israel Deaconess Medical Center (BIDMC) in Boston, Massachusetts, USA. The database offers detailed records on patient demographics, laboratory tests, medications, vital signs, surgical procedures, disease diagnoses, medication management, and follow-up survival status. We focused on the HOSP module, derived from hospital electronic health record (EHR) clinical data. The study utilized the workflow provided by DecisionLinnc (DecisionLinnc Core Team, 2023. DecisionLinnc. Version 1.0, November 2023. Hangzhou, CHN. https://www.statsape.com/). Diagnoses of cirrhosis were extracted from the MIMIC-IV database using the International Classification of Diseases (ICD) codes, leading to the inclusion of 7,277 hospitalized patients diagnosed with cirrhosis. The laboratory results were extracted as the initial measurements taken upon admission. The primary outcome for this external validation study was all-cause mortality within 365 days. In addressing the issue of class imbalance within our dataset, we employed the Synthetic Minority Over-sampling Technique for Nominal and Continuous variables (SMOTE-NC). This sophisticated sampling technique generates new synthetic samples by computing the Euclidean distance between existing variables. Consequently, it improves the representation of the minority class without compromising the structural integrity of the original data. As the database does not contain protected health information, and all patient data is anonymized, written informed consent was waived. We adhered to the guidelines outlined in the ‘Strengthening the Reporting of Observational Studies in Epidemiology’ (STROBE) for observational studies [32].

### Statistical analysis

All the statistical analyses were conducted via STATA v12.0 and R software (version 4.3.1). Categorical variables were analysed via Fisher’s exact test, and continuous variables were analysed via the Wilcoxon rank-sum test. Significance was set at *p* < 0.05. Machine learning methods were implemented in R via packages such as “reticulate”, “caret”, “caretEnsemble”, “randomForest”, “e1071”, “gbm”, “kknn”, and “glmnet”. ROC curves were plotted via the “pROC” package, with the area under the curve (AUC) used to evaluate the diagnostic accuracy of the predictive models. Time-dependent ROC analysis was conducted via the “timeROC” package. Residual analysis and visualisation across multiple algorithmic models were conducted using the “DALEX” package. The model is evaluated for concordance between the predicted probabilities and actual probabilities using calibration curves. The utility of the model is supported by DCA, which reveals significant net benefits across various probability thresholds. The “survival”, “pec”, “rmda”, and “rms” R packages were used to generate concordance curves, DCA plots, C-index plots, and nomograms. Multiple indicator diagnostic ROC curves further validated the diagnostic capacity of the prognostic model relative to single-factor models.

## Results

### Development of the multiple complications prediction model

**Fig. 1:**
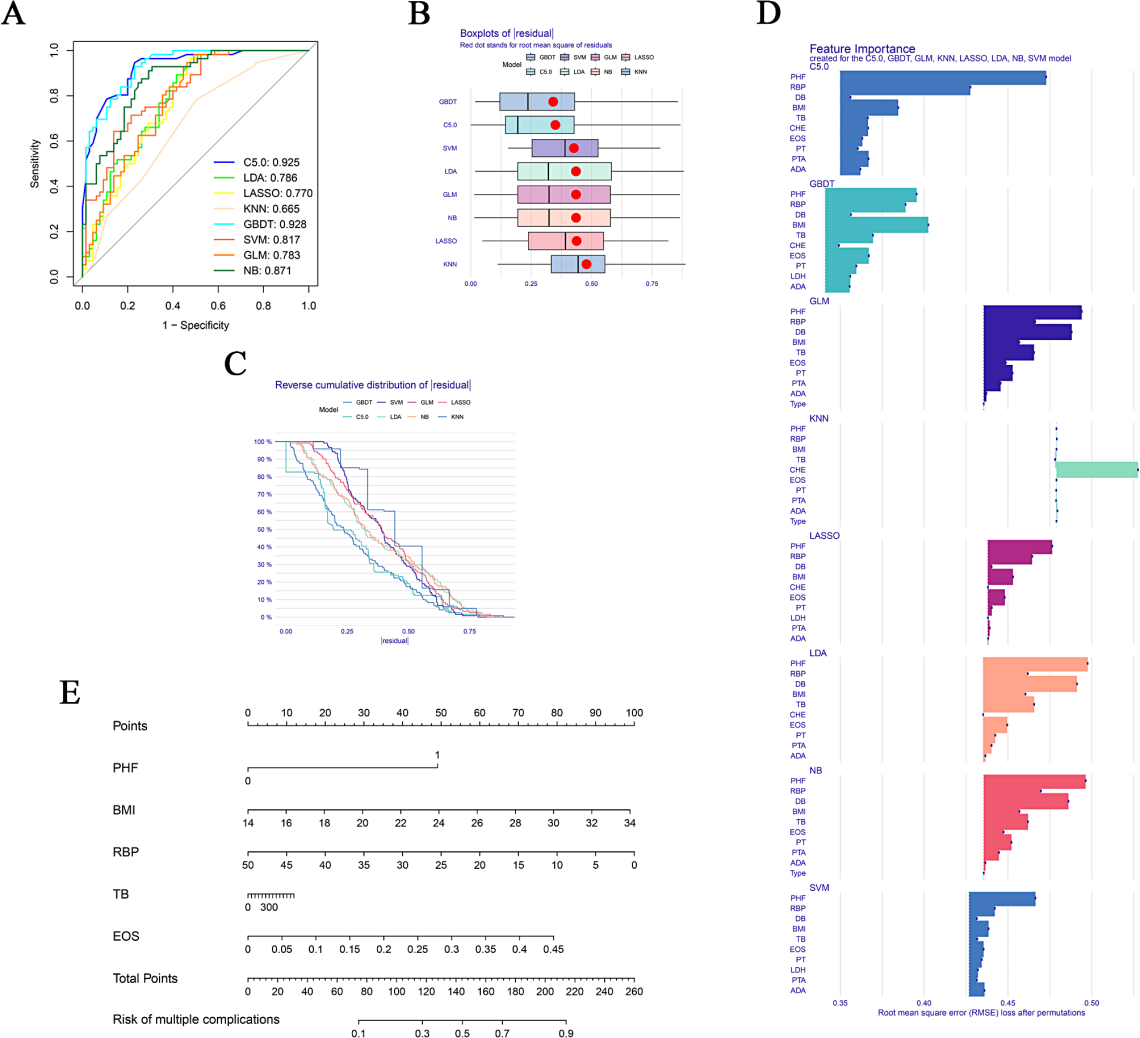
Construction of a multiple complications (three or more) prediction model using eight machine learning methods in hepatitis B-associated cirrhosis (HBC) patients. (**A**) Receiver operating characteristic (ROC) curves for different machine learning models used in predicting multiple complications illustrates the performance of eight machine learning algorithms (C5.0, Linear Discriminant Analysis (LDA), least absolute shrinkage and selection operator (LASSO), k-nearest neighbor (KNN), gradient boosting decision tree (GBDT), support vector machine (SVM), generalized linear model (GLM) and naive bayes (NB)) by displaying their respective area under the curve (AUC) values in predicting multiple complications in HBC patients. (**B**) Residual Box Plots for Machine Learning Models Depicts box plots of residuals for each algorithm to evaluate model fit and variability in residuals across the different machine learning approaches used in the study. (**C**) Cumulative distribution of residuals across machine learning models. This figure provides a comprehensive view of how residuals are distributed cumulatively for each machine learning model, offering insights into the models’ accuracy and prediction errors. (**D**) According to importance scores, the GBDT model identified the five most significant risk factors, including post-hospitalisation fever (PHF), body mass index (BMI), retinol binding protein (RBP), total bilirubin (TB), and eosinophils (EOS). (**E**) A nomogram for predicting the occurrence of multiple complications in HBC patients. To utilise the nomogram, select a distinct value on each variable’s axis and draw a vertical line to ascertain the point’s value. The total points, located at the ‘Total Points’ axis, helps in deducing the likelihood of multiple complications’ occurrence in HBC patients by drawing a line downwards to the corresponding probability

Initially, 57 risk variables were statistically analysed. Categorial variables were examined via Fisher’s exact test, and continuous variables were examined via the Wilcoxon rank-sum test. The variables significantly associated with the onset of multiple complications (*p* < 0.05) included posthospitalisation fever (PHF), BMI, retinol binding protein (RBP) levels, total bilirubin (TB) levels, direct bilirubin (DB), eosinophils (EOS), cholinesterase (CHE), lactate dehydrogenase (LDH), adenosine deaminase (ADA), prothrombin time (PT), and prothrombin time activity (PTA), which were used for model development (Table 2). The machine learning algorithms employed included C5.0, LDA, LASSO, KNN, GBDT, SVM, GLM, and NB. The ROC curves for these methods (Fig. 1A) presented the following AUC values: C5.0 = 0.925, LDA = 0.786, LASSO = 0.770, KNN = 0.665, GBDT = 0.928, SVM = 0.817, GLM = 0.783, and NB = 0.871. Box plots for residuals and the cumulative distribution of residuals (Fig. 1B and C) revealed that the GBDT model, with an AUC of 0.928, exhibited the best fit according to the ROC AUC and residual analysis. A risk factor importance analysis conducted within the GBDT framework identified key predictors, including PHF, BMI, RBP, TB, and EOS (Fig. 1D). A nomogram based on these predictors effectively delineated the risk probabilities for multiple complications in HBC patients (Fig. 1E).

**Table 2:** Risk variables selected for multiple complications prediction model

Variables	Complications < 3	Complications ≥ 3	*p*-value
N	65	56	
Gender			1.00
Woman	16 (25%)	13 (23%)	
Male	49 (75%)	43 (77%)	
HBVFH			1.00
No	50 (77%)	44 (79%)	
Yes	15 (23%)	12 (21%)	
EtOH			0.75
No	60 (92%)	50 (89%)	
Yes	5 (8%)	6 (11%)	
SH			0.73
No	61 (94%)	51 (91%)	
Yes	4 (6%)	5 (9%)	
HCC			0.28
No	38 (58%)	27 (48%)	
Yes	27 (42%)	29 (52%)	
HTN			0.62
No	56 (86%)	46 (82%)	
Yes	9 (14%)	10 (18%)	
DM			0.22
No	57 (88%)	44 (79%)	
Yes	8 (12%)	12 (21%)	
PE			0.83
No	16 (25%)	12 (21%)	
Yes	49 (75%)	44 (79%)	
SV			0.43
No	43 (66%)	41 (73%)	
Yes	22 (34%)	15 (27%)	
PHF			< 0.001*
No	47 (72%)	22 (39%)	
Yes	18 (28%)	34 (61%)	
vomiting			0.58
No	38 (58%)	36 (64%)	
Yes	27 (42%)	20 (36%)	
AVT			0.54
No	7 (11%)	4 (7%)	
Yes	58 (89%)	52 (93%)	
HBVDNA			0.45
Negative	26 (40%)	18 (32%)	
Positive	39 (60%)	38 (68%)	
Age (years, median IQR)	55.0 (46.0, 61.0)	54.5 (45.0, 64.0)	0.48
BMI (median IQR)	22.3 (20.7, 25.2)	24.0 (22.0, 25.4)	0.027*
WBC (10^9/L, median IQR)	4.3 (2.9, 6.2)	4.5 (2.9, 6.5)	0.91
NEU (10^9/L, median IQR)	2.8 (1.7, 4.3)	2.8 (1.5, 4.6)	0.89
NLR (median IQR)	2.9 (1.8, 4.6)	2.7 (1.6, 4.5)	0.93
HB (g/L, median IQR)	123.0 (107.0, 138.0)	120.0 (108.0, 133.0)	0.50
PLT (10^9/L, median IQR)	65.0 (45.0, 94.0)	67.0 (47.0, 106.5)	0.66
LYM (10^9/L, median IQR)	1.0 (0.8, 1.3)	0.9 (0.7, 1.3)	0.42
PLR (median IQR)	66.3 (45.2, 97.4)	74.9 (55.9, 106.9)	0.14
RBC (10^12/L, median IQR)	3.7 (3.2, 4.3)	3.6 (3.2, 4.0)	0.56
HCT (%,median IQR)	35.7 (30.5, 40.7)	35.1 (31.0, 38.7)	0.47
MONO (10^9/L, median IQR)	0.3 (0.2, 0.5)	0.3 (0.3, 0.6)	0.48
EOS (10^9/L, median IQR)	0.0 (0.0, 0.1)	0.1 (0.0, 0.1)	0.043*
TB (µmol/L, median IQR)	22.0 (14.1, 35.9)	25.5 (20.2, 49.7)	0.023*
DB (µmol/L, median IQR)	9.8 (5.4, 13.6)	11.9 (8.4, 25.9)	0.008*
ALT (U/L, median IQR)	36.2 (21.8, 62.3)	43.0 (30.1, 66.8)	0.32
AST (U/L, median IQR)	40.7 (26.4, 78.1)	55.7 (34.2, 93.5)	0.051
ALB (g/L, median IQR)	33.8 (28.3, 39.3)	32.0 (29.0, 35.9)	0.10
GLO (g/L, median IQR)	30.6 (26.1, 34.7)	32.3 (28.5, 36.8)	0.24
GGT (U/L, median IQR)	57.3 (27.0, 130.0)	79.0 (35.2, 170.4)	0.085
CHE (U/L, median IQR)	3884.0 (2466.0, 5710.0)	3401.5 (2223.0, 4012.0)	0.034*
ALP (U/L, median IQR)	86.1 (63.7, 129.6)	114.3 (76.9, 153.3)	0.062
LDH (U/L, median IQR)	202.0 (153.0, 235.0)	223.0 (178.5, 273.0)	0.032*
TBA (µmol/L, median IQR)	21.6 (6.9, 42.1)	29.5 (12.1, 94.1)	0.061
Lactate (mmol/L, median IQR)	2.6 (2.2, 3.0)	2.7 (2.2, 3.0)	0.60
RBP (mg/L, median IQR)	22.7 (16.9, 30.0)	17.8 (11.8, 23.5)	0.002*
AFU (U/L, median IQR)	18.0 (14.0, 24.0)	20.0 (15.5, 25.5)	0.16
MAO (U/L, median IQR)	4.5 (3.1, 6.5)	5.3 (4.1, 6.6)	0.15
ADA (U/L, median IQR)	22.4 (16.1, 29.2)	26.9 (20.9, 32.6)	0.026*
B2M (mg/ml, median IQR)	3.0 (2.3, 3.8)	2.8 (2.0, 4.2)	0.63
serum potassium (mmol/L, median IQR)	3.8 (3.6, 4.1)	3.8 (3.6, 4.2)	0.87
serum sodium (mmol/L, median IQR)	140.6 (138.1, 142.7)	139.4 (136.9, 141.8)	0.15
serum calcium (mmol/L, median IQR)	2.1 (1.9, 2.2)	2.0 (1.9, 2.1)	0.29
serum ferritin (umol/L, median IQR)	20.8 (11.4, 30.1)	18.5 (8.4, 27.7)	0.16
serum phosphate (mmol/L, median IQR)	1.0 (0.9, 1.1)	1.0 (0.8, 1.1)	0.89
BUN (mmol/L, median IQR)	5.3 (4.7, 6.5)	6.1 (4.5, 8.3)	0.080
Cr (µmol/L, median IQR)	68.0 (58.0, 76.0)	70.5 (56.7, 97.0)	0.18
PT (second, median IQR)	14.4 (13.4, 15.5)	15.4 (14.1, 17.5)	0.020*
APTT (second, median IQR)	33.8 (30.2, 40.6)	38.2 (33.3, 43.6)	0.072
TT (second, median IQR)	21.1 (19.6, 23.2)	21.0 (20.0, 23.0)	0.97
Fib (g/L, median IQR)	1.6 (1.4, 2.3)	1.8 (1.4, 2.5)	0.53
AT3 (mg/dl, median IQR)	75.5 (57.5, 84.4)	65.8 (48.9, 85.8)	0.17
PTA (%,median IQR)	62.6 (53.8, 71.6)	55.9 (48.0, 65.9)	0.046*
AFP (ng/ml, median IQR)	47.3 (6.6, 239.7)	15.3 (4.1, 239.7)	0.39

Note. *P < 0.05. Categorical variables were analyzed using Fisher’s exact test, and continuous variables were evaluated with the Wilcoxon rank-sum test to calculate P-values

### Internal validation and performance evaluation of the prediction model

**Fig. 2:**
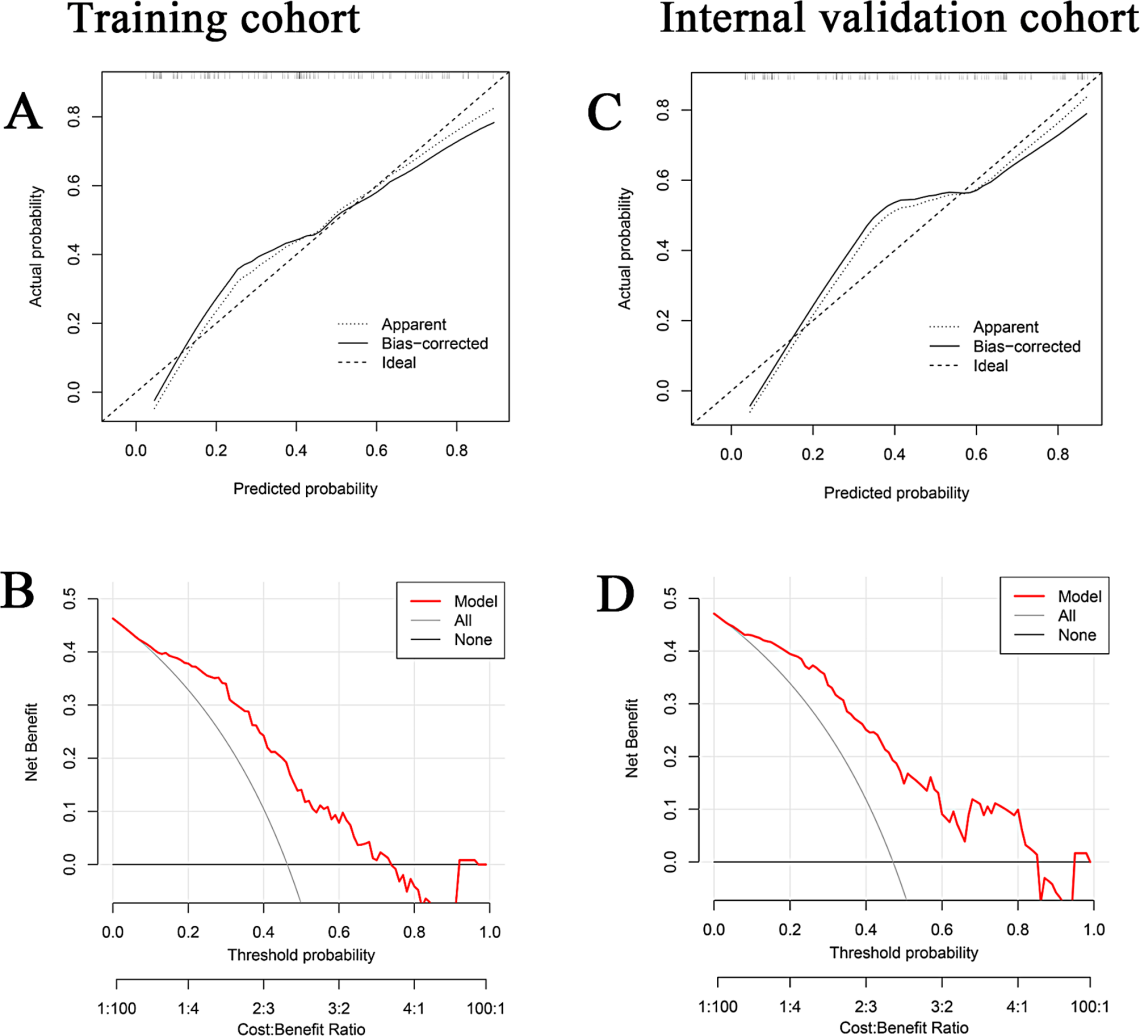
Calibration and decision curve analysis (DCA) for the GBDT prediction model. (**A**) Calibration curve illustrating good fit of the nomogram. the x-axis denotes predicted probabilities, representing the likelihood of event occurrence estimated by the prediction model, ranging from 0 to 100%. The y-axis represents actual probabilities, indicating the actual incidence rate in patients. The black solid line represents the prognostic performance of the nomogram. (**B**) DCA evaluating the clinical applicability of the nomogram in the training cohort. The y-axis represents net benefit, with thin lines hypothesising the occurrence of multiple complications in all patients, and thicker lines assuming no occurrence in any patient, with the red line representing the nomogram for multiple complications in HBC patients. (**C**) Calibration curve analysis in the internal validation cohort. (**D**) DCA in the internal validation cohort

Subsequently, the same machine learning analysis was conducted on 121 patients, revealing the AUC values for the C5.0, LDA, LASSO, KNN, GBDT, SVM, GLM, and NB models to be 0.985, 0.857, 0.812, 0.785, 0.999, 0.915, 0.856, and 0.915 respectively (Supplementary Fig. 1A). Residual analysis (Supplementary Fig. 1B, C) also indicated that the GBDT model had the lowest residual values, thereby confirming that the GBDT model is the optimal model for predicting multiple complications in HBC patients. This internal validation corroborated the GBDT model as the superior predictive framework among all the models constructed. Both the training and internal validation cohorts demonstrated strong predictive accuracy and clinical utility, as confirmed by calibration and DCAs (Fig. 2A-D).

### Construction of the survival prognostic model for HBC patients

**Fig. 3:**
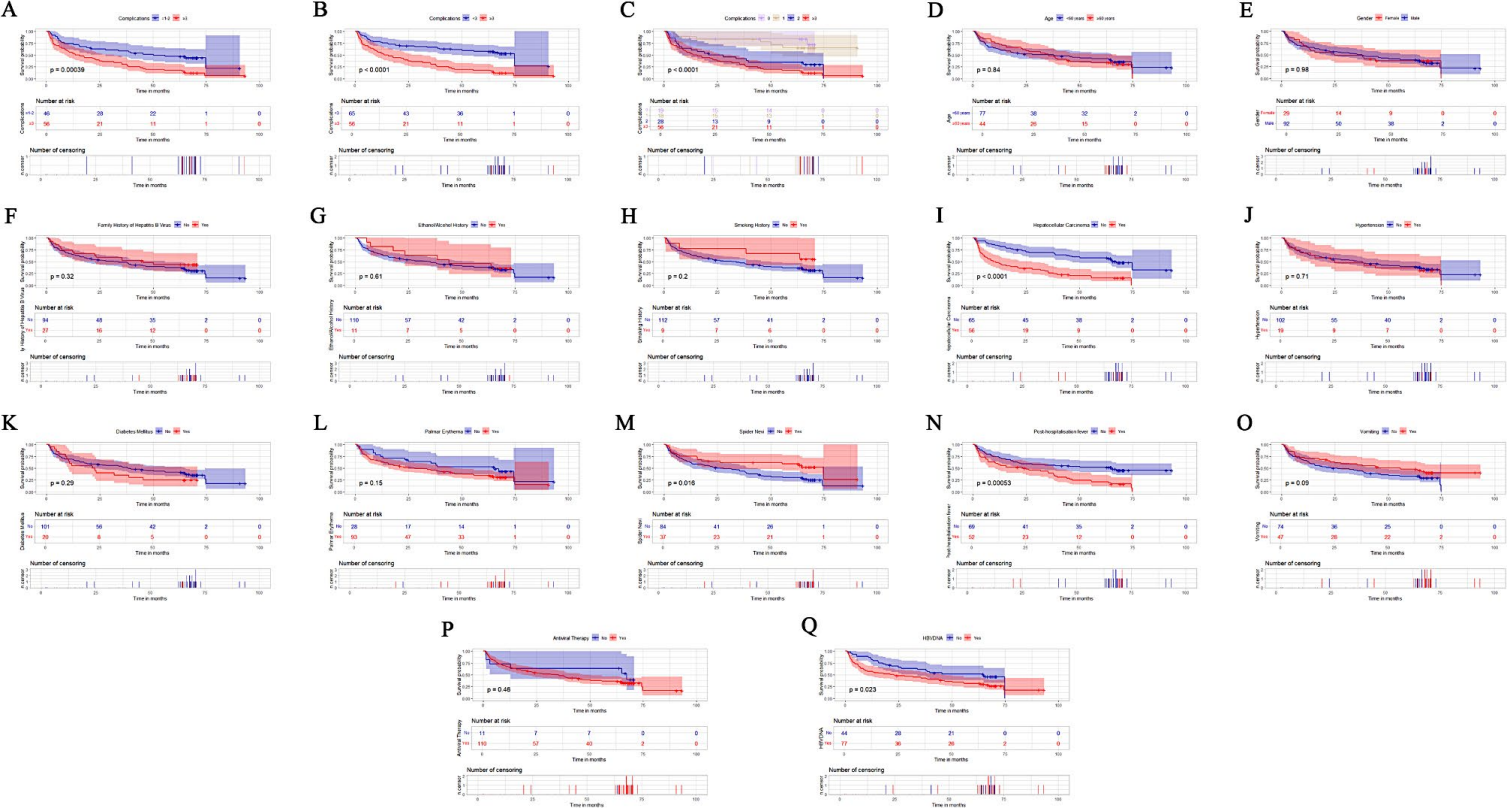
Kaplan-Meier curves analyzing the occurrence of multiple complications and key patient characteristics on prognosis in HBC patients. (**A**) Patients with multiple complications exhibit a significantly poorer overall survival (OS) compared to those with 1–2 complications. (**B**) Compared to patients with fewer than three complications, those with multiple complications show considerably lower OS rates. (**C**) Multiple comparison analysis using Kaplan-Meier curves demonstrates that compared to no complications, one complication, or two complications, patients with multiple complications experience the poorest OS. Kaplan-Meier survival analysis was performed to assess variations in OS among different subgroups, which include age (**D**), gender (**E**), family history of hepatitis B virus (**F**), history of alcohol consumption (**G**), smoking history (**H**), hepatocellular carcinoma (**I**), hypertension (**J**), diabetes mellitus (**K**), palmar erythema (**L**), spider naevi (**M**), postoperative fever (**N**), vomiting (**O**), antiviral treatment with nucleoside analogues (**P**), and HBV DNA levels (**Q**)

Kaplan-Meier survival analysis demonstrated that HBC patients with three or more combined complications had significantly lower OS compared to HBC patients with one or two complications (*P* < 0.001, Fig. 3A). Furthermore, their OS was also significantly lower than that of HBC patients with fewer than three complications (*P* < 0.001, Fig. 3B). Multiple comparison analysis further highlighted that patients with three or more complications had the worst survival outcomes (*P* < 0.001, Fig. 3C). Accordingly, we conducted a subgroup analysis to evaluate prognostic differences among key demographic characteristics. The results are illustrated in Fig. 3D-Q. Notably, significant prognostic differences were observed among the HBC patient population specifically within the subgroups stratified by HCC, spider nevi, PHF, and HBVDNA. This finding raises the following question: What is the impact of having more than three complications on the OS of patients with HBC? We included multiple complications as an independent variable in the survival analysis to assess the prognostic significance of multiple complications in patients with HBC.

**Fig. 4:**
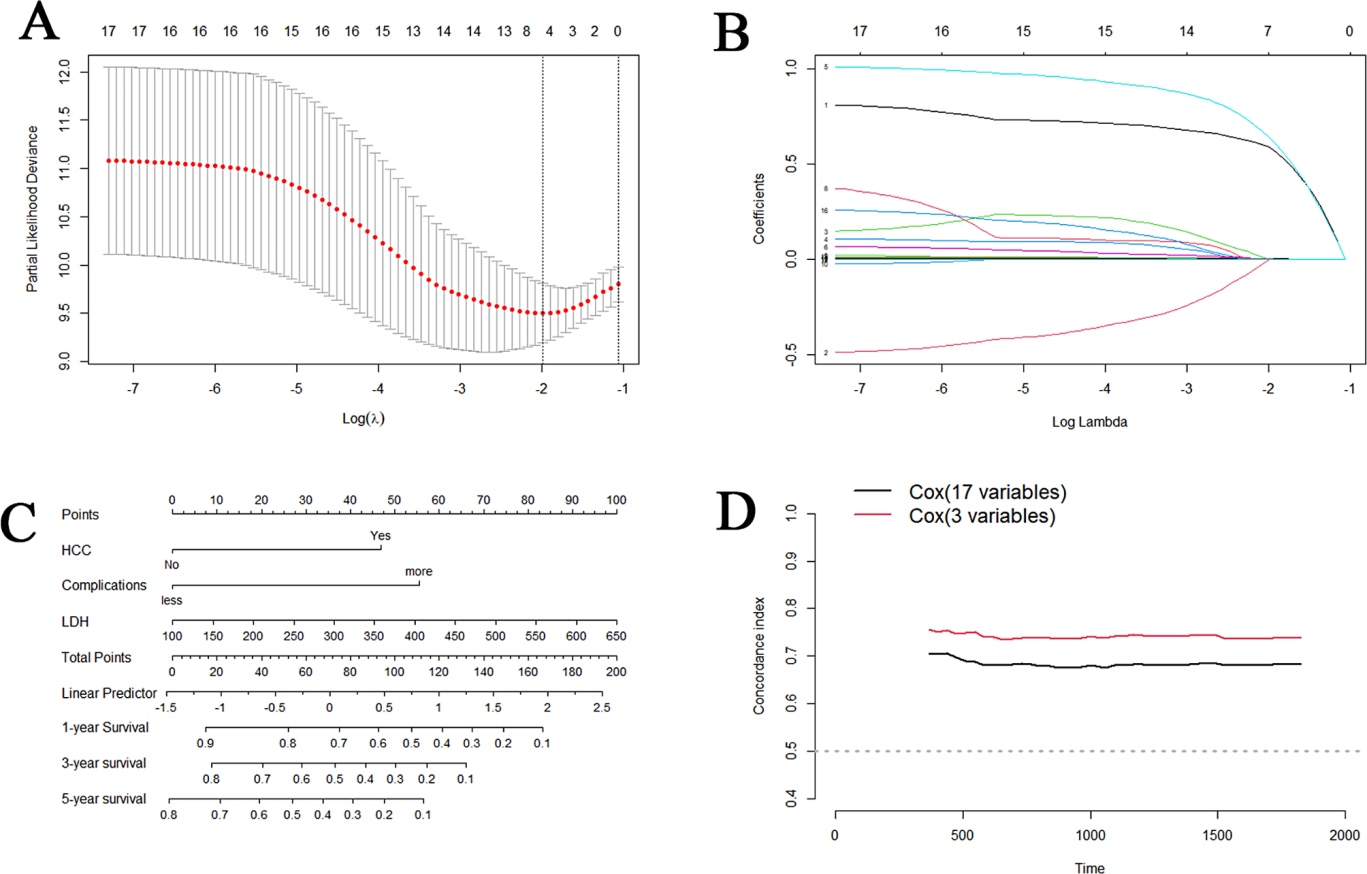
Selection of risk variables using the least absolute shrinkage and selection operator (LASSO)-Cox regression model. (**A**) LASSO coefficient curves for 17 features related to HBC patient survival. (**B**) Tri-fold cross-validation for optimal parameters (lambda) selection in the LASSO model, with the partial likelihood deviance curves plotted against log(lambda). Dotted vertical lines at the optimal values using minimum criteria and 1 SE (1-Standard Error criterion) are shown. (**C**) Constructing a nomogram based on the results of a multivariable cox regression analysis. (**D**) The C-index compared with a model of 17 risk variables. HCC, Hepatocellular Carcinoma; LDH, lactic dehydrogenase

Univariable Cox regression analysis of 58 variables (including multiple complication variables) identified 17 statistically significant prognostic factors (*p* < 0.05). LASSO regression of these variables revealed four pertinent risk factors: HCC, multiple complications, LDH, and alpha-l-fucosidase (AFU) (Fig. 4A, B). Multivariate Cox regression confirmed these three factors—HCC, multiple complications, and LDH—as independent prognostic factors for OS in HBC patients and were used to construct a prognostic survival model nomogram (Table 3; Fig. 4C). Compared with a preidentified 17-variable model, the new prognostic model exhibited a superior c-index, indicating enhanced predictive power (Fig. 4D).

**Table 3:** Multivariable cox regression analysis for identified independent prognostic factors

characteristics	HR	95%CI	*P* value
HCC	2.506	1.572–3.994	< 0.001
LDH	1.004	1.002–1.006	< 0.001
multiple complications	3.063	1.908–4.915	< 0.001
AFU	1.016	1.000-1.033	0.053

Note. HR, hazard ratio; CI, confidence interval

### Prognostic model performance evaluation

**Fig. 5:**
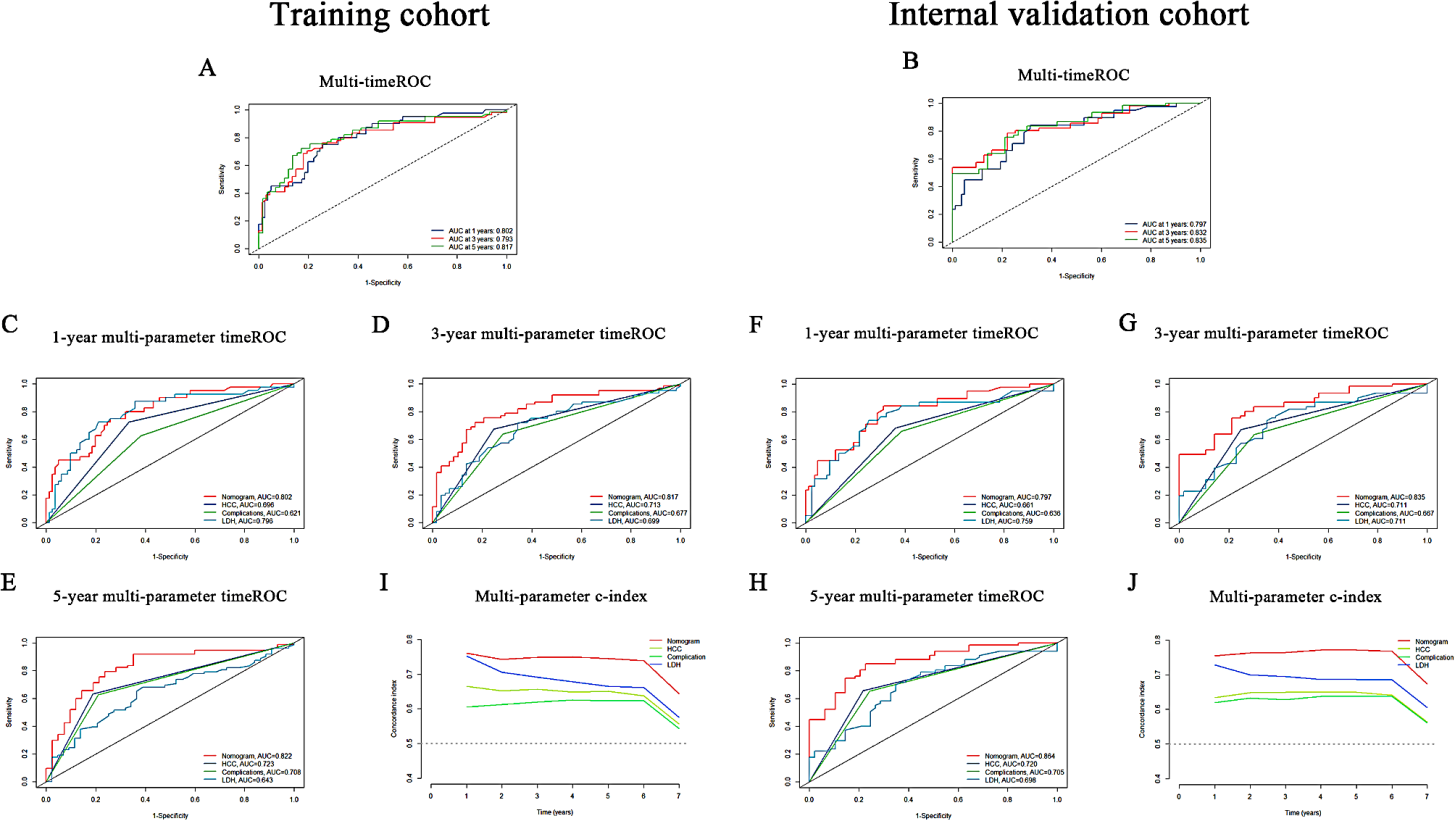
ROC curves predicting 1-, 3-, and 5-year survival for the survival prognostic model. (**A**) Time-dependent ROC (timeROC) curves at 1, 3, and 5 years in the training cohort. (**B**) TimeROC curves at 1, 3, and 5 years in the internal validation cohort. (**C**-**E**) Multi-parameter diagnostic timeROC at 1, 3, and 5 years In the training cohort. Comparison of the predictive accuracy of the survival prognostic model and models based on single risk factors (hepatocellular carcinoma (HCC), multiple complications, lactate dehydrogenase (LDH) over 1-, 3-, and 5-year intervals in the training cohort. (**F**-**H**) Multi-parameter diagnostic timeROC at 1, 3, and 5 years in the internal validation cohort. (**I**) Multi-parameter c-index in the training cohort. Comparison of C-indices between the survival prognostic model and single risk factor models in the training cohort. (**J**) Multi-parameter c-index in the internal validation cohort. Comparison of C-indices between the survival prognostic model and single risk factor models in the internal validation cohort

**Fig. 6:**
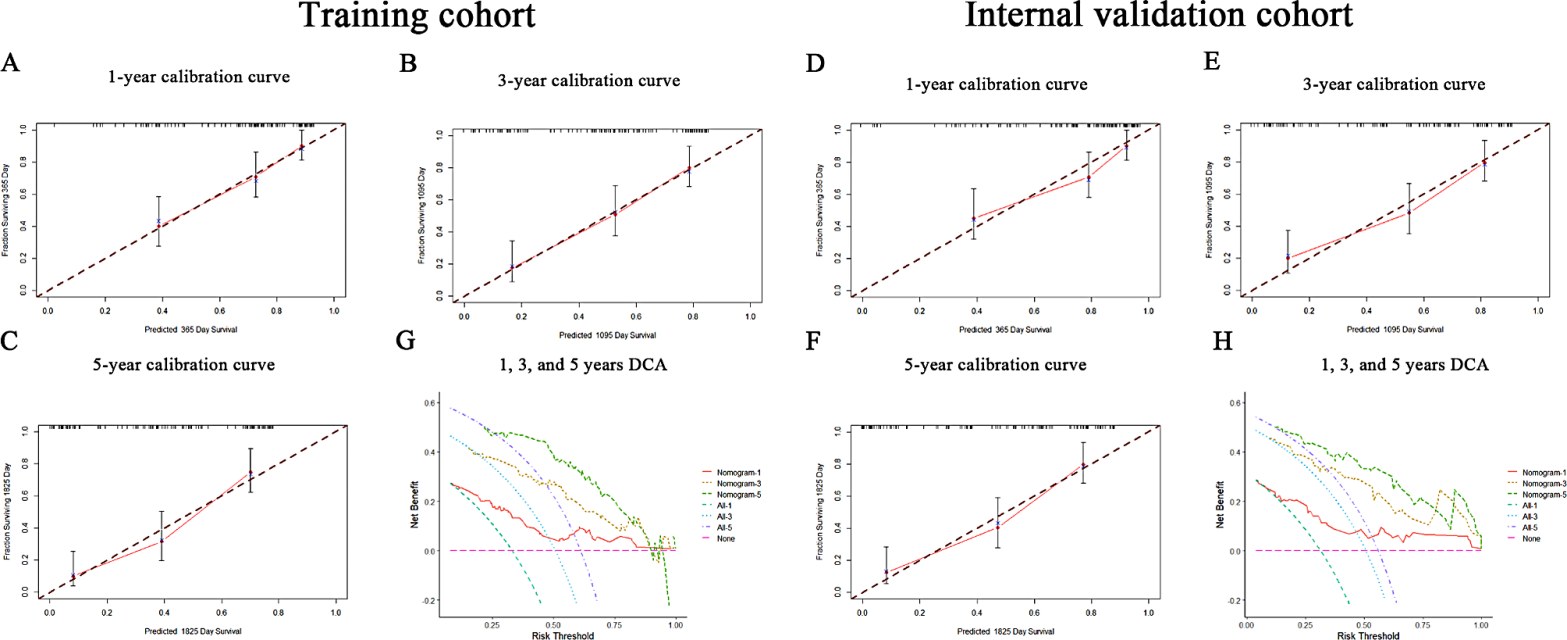
Calibration curve analysis and DCA for the survival prognostic model. (**A**-**C**) Calibration curves for the survival prognostic model at 1-, 3-, and 5-year intervals in the training cohort. (**D**-**F**) Calibration curves for the survival prognostic model at 1-, 3-, and 5-year intervals in the internal validation cohort. (G) DCA for the survival prognostic model in the training cohort. (H) DCA for the survival prognostic model in the internal validation cohort

**Fig. 7:**
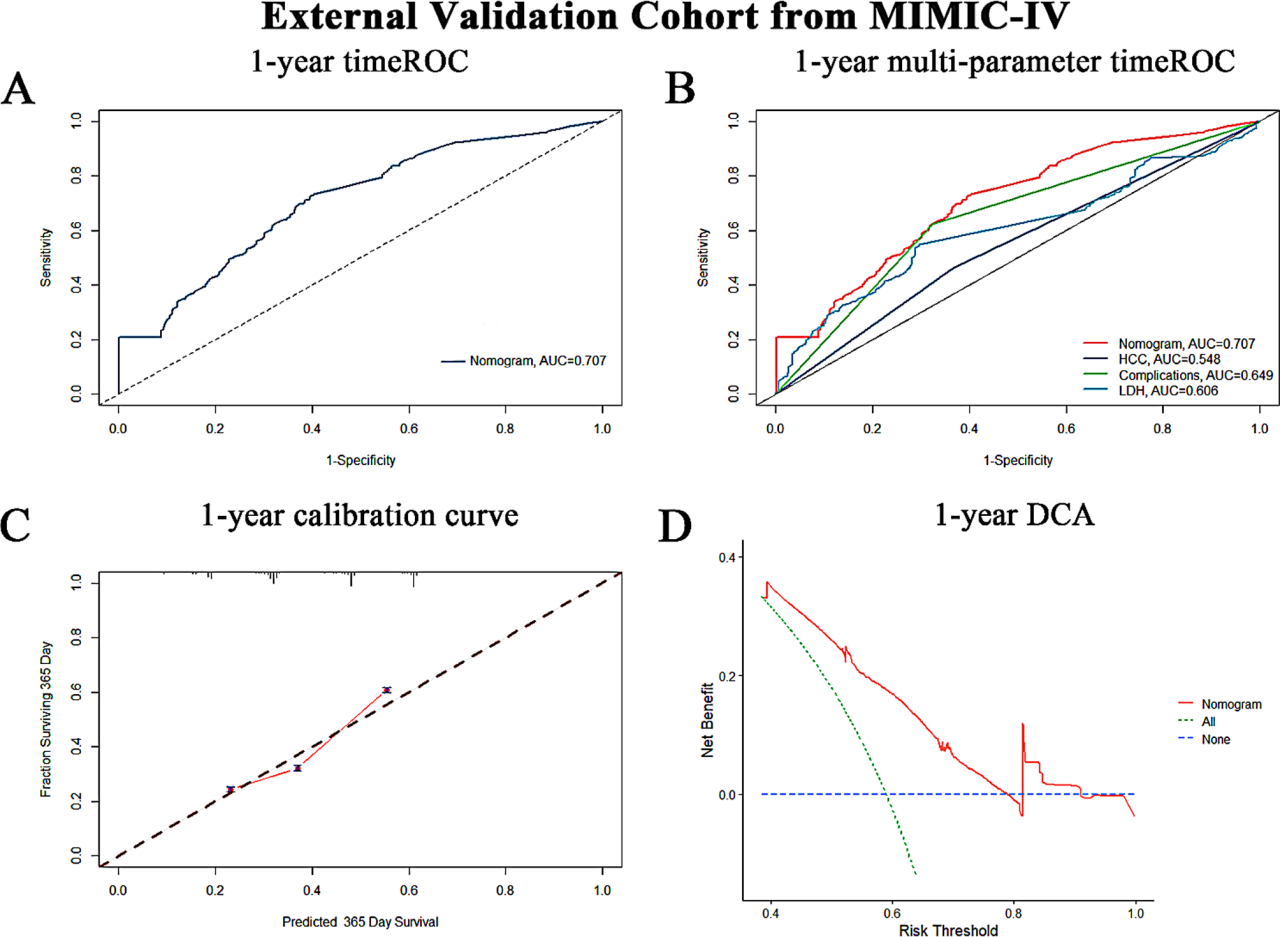
Evaluation of the Prognostic Model Using the Medical Information Mart for Intensive Care IV (MIMIC-IV) Cohort. (**A**) ROC curve depicting the 1-year mortality prediction performance of the developed risk score model in patients with cirrhosis from the MIMIC-IV cohort. The AUC is 0.707. (**B**) Comparison of 1-year time-dependent ROC AUCs for different univariate models: the prognostic model (AUC = 0.707), HCC model (AUC = 0.548), complications model (AUC = 0.649), and LDH model (AUC = 0.606). The prognostic model shows superior performance. (**C**) Calibration curve assessing the agreement between predicted probabilities and observed outcomes of 1-year mortality risk, indicating good calibration of the prognostic model. (**D**) DCA demonstrating the clinical utility and net benefit of the prognostic model across various threshold probabilities, supporting its application in clinical practice for individualized risk assessment in cirrhotic patients

**Fig. 8:**
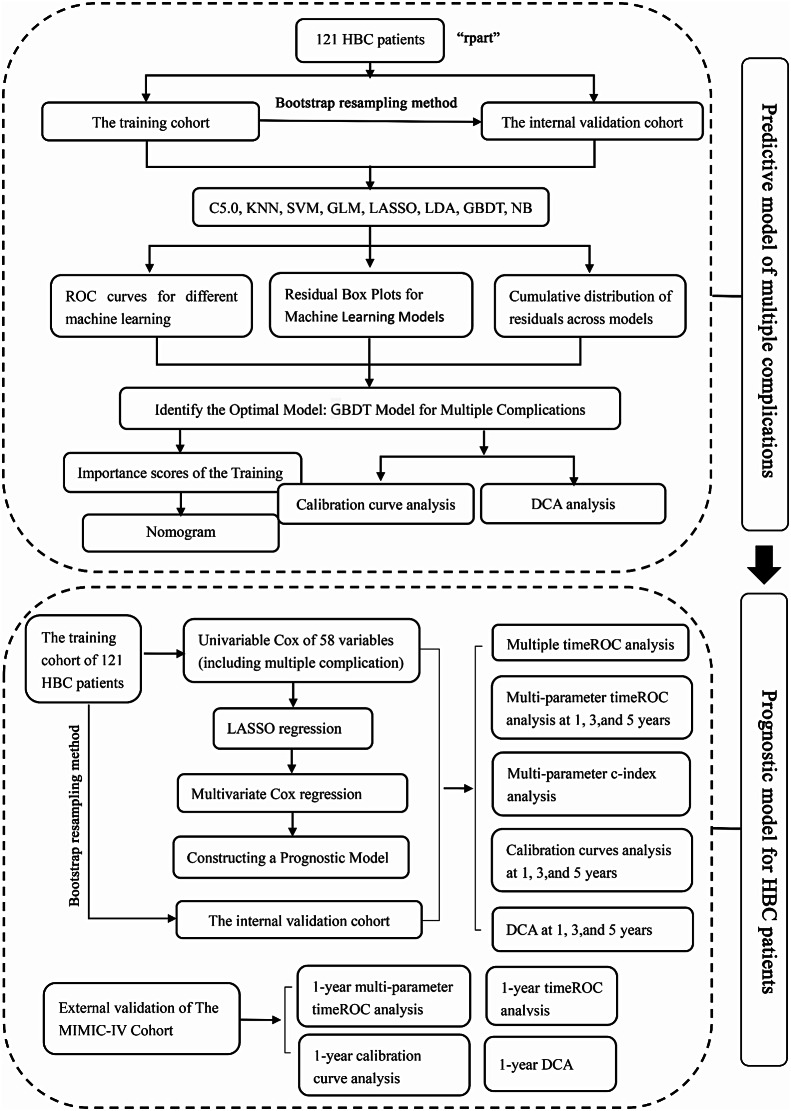
The flowchart of the study

The three-factor prognostic model, involving HCC, complications, and LDH levels, was internally validated using bootstrap resampling, showing consistent metrics in both training and validation cohorts. TimeROC analyses demonstrated AUC values of 0.802, 0.793, and 0.817 at 1, 3, and 5 years in the training cohort (Fig. 5A), and 0.797, 0.832, and 0.835 in the validation cohort (Fig. 5B). The increasing 5-year AUCs indicate the model’s growing predictive accuracy and reliability for long-term patient management, aiding in optimising management plans, treatment regimens, and resource allocation for high-risk patients. To assess the model’s performance, we compared the comprehensive multivariable model with univariate prognostic models based on HCC, complications, and LDH. These specific univariate prognostic models are referred to as the HCC model, complications model, and LDH model, respectively. These terms refer to specific univariate prognostic models that evaluate HCC, multiple complications, and LDH as independent variables impacting the survival outcomes of patients with HBC. In the training cohort, the 1-year AUCs were 0.802, 0.696, 0.621, and 0.796, respectively; at 3 years, 0.817, 0.713, 0.677, and 0.699; and at 5 years, 0.822, 0.723, 0.708, and 0.643. In the validation cohort, 1-year AUCs were 0.797, 0.661, 0.636, and 0.759; 3-year AUCs were 0.835, 0.711, 0.667, and 0.711; and at 5 years, 0.864, 0.720, and 0.698 (Fig. 5C-H). These results endorse the model’s robust performance across various timelines and factors. The prognostic model exhibited the highest concordance index in both cohorts, as shown in Fig. 5I and J. Calibration curves for 1-, 3-, and 5-year predictions demonstrated robust model concordance (Fig. 6A-F). Decision curve analysis confirmed the model’s clinical utility, with significant net gain (Fig. 6G-H). External validation using the MIMIC-IV database showed a 1-year ROC AUC of 0.707, outperforming univariate models of HCC, complications, and LDH (0.548, 0.649, 0.606) (Fig. 7A-B). Calibration curves indicated good prediction accuracy (Fig. 7C), and DCA affirmed its clinical utility (Fig. 7D). Figure 8 illustrates the flowchart of our study.

## Discussion

Cirrhosis, despite its significant global prevalence and disease burden, remains underrecognised compared to conditions like heart failure and chronic kidney disease [33]. Complications such as infections, ascites, hepatorenal syndrome, variceal bleeding, SBP, and hepatic encephalopathy significantly impact patient quality of life and increase mortality rates [34]. Ascites develops in around 60% of compensated cirrhosis cases within 10 years, with a three-year mortality rate of up to 50% [35]. Variceal bleeding poses a high mortality risk, affecting 40% of compensated and 85% of decompensated cirrhosis patients [36–38]. Hepatic encephalopathy severely affects prognosis, contributing to higher hospitalisation rates, costs, and diminished quality of life [39–41]. Acute kidney injury (AKI) also frequently occurs with lower survival rates [42, 43]. Cirrhotic patients have an elevated risk of infections, including SBP, with a global prevalence of 17.12% and a 30.61% associated mortality rate [44]. Additionally, common infections include urinary tract infections, pneumonia, cellulitis, and bacteremia, with pneumonia linked to multiorgan failure [45–47]. Despite extensive studies on cirrhosis complications, research on prediction and prognostic systems addressing multiple complication incidents in HBC is limited. This study developed and validated a predictive model for HBC complications using advanced machine learning techniques, including C5.0, LDA, LASSO, KNN, GBDT, SVM, GLM, and NB. The GBDT model excelled, showing superior performance and reliability confirmed by bootstrap resampling and external MIMIC-IV database validation. Key risk factors identified for complications include PHF, BMI, RBP, TB, and EOS.

Low to moderate fevers in cirrhosis may stem from protein byproducts of hepatocyte necrosis affecting thermoregulation, indicating disease progression. High fevers often signal complications [48, 49]. BMI, a marker for obesity, is a crucial factor in HBC progression, with high BMI linked to worsened liver fibrosis and impeded fibrosis improvement [50, 51]. RBP, produced by hepatocytes, reflects liver storage and damage, offering prognostic value in liver disease. Although data on RBP in HBC is limited, it has been identified as a significant marker for hepatic encephalopathy [52, 53]. Total bilirubin TB is crucial for assessing jaundice and liver function. It predicts early hepatorenal syndrome and mortality in variceal bleeding [54–56]. Eosinophils play varied roles in cirrhosis. A low eosinophil count predicts in-hospital mortality among cirrhosis patients with severe systemic inflammatory response syndrome (SIRS) [57]. Cirrhosis involves systemic inflammation through immune deficiency and hyperactivation. Decompensated cirrhosis and acute-chronic liver failure (ACLF) differ: ACLF leads to eosinophil depletion, while decompensated cirrhosis shows increased eosinophils [58–63]. Ascites results from proinflammatory cytokine-induced vasodilation and renal damage via pathogen-associated molecular patterns (PAMPs) and damage-associated molecular patterns (DAMPs), correlating with circulating proinflammatory triggers [59–61]. The emergence of clinical complications is also associated with systemic inflammation and oxidative stress, which have been described as potential mechanisms of HE [64, 65]. Eosinophil levels have been notably elevated in cirrhotic patients, reflecting systemic inflammation [66]. These insights underline the complexity of cirrhosis-related inflammation and immune changes, illustrating the interplay between systemic factors and clinical complications in disease progression.

Our study confirms PHF, BMI, RBP, TB, and EOS as significant predictive risk factors for multiple complications in HBC, supporting their documented roles in liver disease progression. We developed a nomogram to predict these complications, providing a valuable tool for clinical application. Our survival analysis indicated that patients with multiple complications had significantly lower survival rates compared to those with fewer complications. Through univariate Cox regression, LASSO regression, and multivariate Cox regression, we identified independent prognostic markers: HCC, multiple complications, and LDH. This prognostic model surpasses traditional models in terms of the concordance index and area under the curve, across different timelines. HCC is a major cause of liver-related mortality, particularly in HBC patients, as highlighted by long-term studies in South China [67, 68]. Elevated LDH levels, a marker of hepatic cellular necrosis, are independent predictors of survival in HBC and subacute liver failure, and play a prognostic role in HBV-related HCC [69, 70]. The potential of LDH as a prognostic factor for OS in HBC patients remains underexplored; however, our study suggests it may enhance survival predictions at 1, 3, and 5 years via nomograms. Research is limited on the long-term impact of multiple complications on HBC prognosis, but our findings align with Bhattarai’s evaluation of complications in decompensated cirrhosis patients [71]. Our study identifies multiple complications as independent prognostic factors for OS, with implications for healthcare utilisation, as the presence of multiple complications significantly increases costs and hospital stays [72]. In ACLF, multiple concurrent complications are critical indicators of exacerbation and mortality risk [73].

Our model for predicting multiple complications and prognosis in HBC patients may have wider relevance to other liver diseases, though direct applicability remains unreported. Key variables identified—fever, BMI, RBP, total bilirubin, eosinophil count, HCC, and LDH—are potentially applicable to broader liver conditions. Fever and bilirubin, for example, feature in prognostic tools for alcoholic hepatitis [74]. BMI is already a recognized predictor for non-alcoholic fatty liver disease (NAFLD) and metabolic dysfunction-related liver diseases [75–77]. Elevated RBP4 serves as a potential marker for early NAFLD diagnosis [78], and total bilirubin is pivotal in multiple liver disease models, including as a predictor for advanced fibrosis and cholangiocarcinoma [79–84]. EOS is crucial for assessing drug-induced liver injury [85]. In our prognostic model for HBC patients, incorporating multiple complications, HCC, and LDH is novel. Multiple complications are known as mortality risk factors in ACLF [73]. HCC’s association with NAFLD is evident through mechanisms promoting hepatic inflammation and carcinogenesis [86–88]. LDH predicts mortality in acute liver failure and is linked with hepatic damage in intrahepatic cholestasis of pregnancy [89–91]. However, applying our model to non-HBC populations might be limited due to different pathophysiological contexts. Future studies should validate and adapt the model for other liver diseases, possibly incorporating specific variables and expanding to diverse populations via multicentre collaborations.

Our model offers clinicians a tool to predict multiple complications and assess their impact on the survival of HBC patients. These capabilities can inform tailored management strategies, enhance healthcare resource utilisation, and improve patient outcomes. By using machine learning, our model prioritises patients based on risk, aiding in more intensive monitoring or early intervention to mitigate complications. The prognostic model highlights risk factors affecting prognosis, helping to identify high-risk populations for early intervention and mortality risk reduction. These models enhance clinical decision-making by translating complex data into actionable insights, promoting proactive HBC management with an emphasis on prevention. However, further refinement is needed to improve predictive accuracy and applicability across diverse populations. Future research will involve multi-centre collaborations and prospective validation to strengthen the model’s practicality and generalisability. While our study provides a framework for predicting complications in HBC, its limitations include the need for validation in larger, diverse cohorts and the retrospective nature of the analysis, which limits assessment of medication and outcomes. External validation with the MIMIC cohort, reflecting varied cirrhosis practices, highlights discrepancies that may affect generalisability, underscoring the need for additional HBC-specific data to confirm the model’s robustness.

## Conclusion

In conclusion, our study constructs and validates two models: one that predicts the occurrence of multiple complications and one that predicts OS in HBC patients. These models not only facilitate the early identification of complication risks using clinically accessible risk factors but also have the potential to assist in clinical interventions aimed at mitigating these complications, thereby potentially enhancing the long-term survival rates of HBC patients. Two nomograms demonstrate increased clinical applicability by providing a clear and interpretable methodology to support clinical decisions, assisting health care professionals, and assisting family members in assessing the long-term prognosis of patients with HBC.

## Electronic supplementary material

Below is the link to the electronic supplementary material.

Supplementary Material 1: Utilisation of machine learning to validate a multiple complications prediction model within an internal validation cohort. (A) Analysis of ROC curves for eight machine learning algorithms in the internal validation cohort. (B) Box plots of residuals for the eight machine learning algorithms in the internal validation cohort. (C) Cumulative distribution plots of residuals for the eight machine learning algorithms in the internal validation cohort.

Supplementary Material 2

Supplementary Material 3

## Data Availability

Data is provided within the manuscript or supplementary information files.

## Abbreviations

ACLF: Acute‒chronic liver failure
ADA: Adenosine deaminase
AFU: Alpha-l-fucosidase
AIC: Akaike Information Criterion
AKI: Acute kidney injury
AUC: Area under the curve
BIDMC: Beth Israel Deaconess Medical Center
BMI: Body mass index
BT: Bacterial translocation
CHE: Cholinesterase
DAMPs: Damage-associated molecular patterns
DCA: Decision curve analysis
EHR: Electronic health record
EASL: European Association for the Study of the Liver
EOS: Eosinophils
GBDT: Gradient boosting decision tree
GLM: Generalised linear model
HBC: Hepatitis B-associated cirrhosis
HBV: Hepatitis B virus
HCC: Hepatocellular carcinoma
HE: Hepatic encephalopathy
ICD: International Classification of Diseases
KNN: k-nearest neighbour
LASSO: Least absolute shrinkage and selection operator
LDA: Linear discriminant analysis
LDH: Lactate dehydrogenase
MIMIC-IV: Medical Information Mart for Intensive Care IV
ML: Machine learning
NAFLD: Non-alcoholic fatty liver disease
NB: Naive Bayes
OS: Overall survival
PAMPs: Pathogen-associated molecular patterns
PHF: Posthospitalisation fever
RBP: Retinol binding protein
ROC: Receiver operating characteristic
SBP: Spontaneous bacterial peritonitis
SD: Standard Deviation
SIRS: Severe systemic inflammatory response syndrome
SMOTE-NC: Synthetic Minority Over-sampling Technique for Nominal and Continuous variables
PT: Prothrombin time
PTA: Prothrombin time activity
STROBE: Strengthening the Reporting of Observational Studies in Epidemiology
SVM: Support vector machine
TB: Total bilirubin
timeROC: Time-dependent ROC
