# Supplementary figures and images for: Identification of multiple complications as independent risk factors associated with 1-, 3-, and 5-year mortality in hepatitis B-associated cirrhosis patients
Source: BMC Infect Dis. 2025 Feb 1;25:151. doi: 10.1186/s12879-025-10566-6 (PMC11786570; doi:10.1186/s12879-025-10566-6)

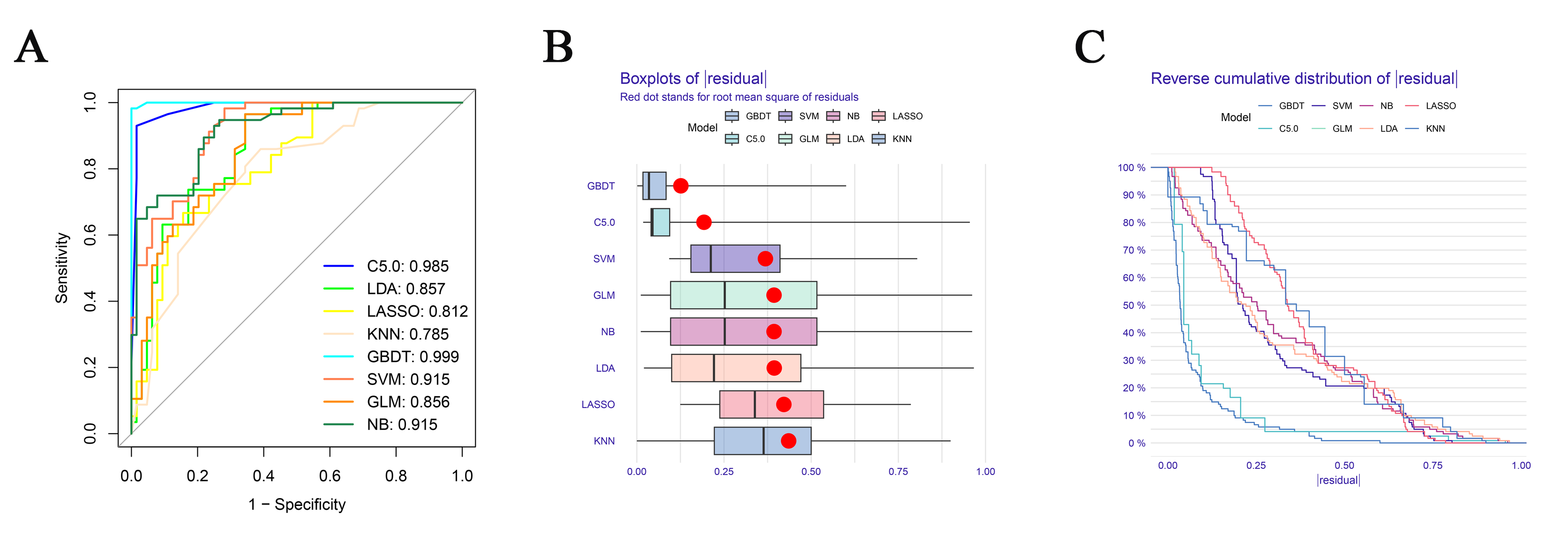

Supplement: Supplementary file 1 — Supplementary Material 1: Utilisation of machine learning to validate a multiple complications prediction model within an internal validation cohort. (A) Analysis of ROC curves for eight machine learning algorithms in the internal validation cohort. (B) Box plots of residuals for the eight machine learning algorithms in the internal validation cohort. (C) Cumulative distribution plots of residuals for the eight machine learning algorithms in the internal validation cohort. [file 12879_2025_10566_MOESM1_ESM.tif]
