# Supplementary material for: Identification of multiple complications as independent risk factors associated with 1-, 3-, and 5-year mortality in hepatitis B-associated cirrhosis patients
Source: BMC Infect Dis. 2025 Feb 1;25:151. doi: 10.1186/s12879-025-10566-6 (PMC11786570; doi:10.1186/s12879-025-10566-6)
Supplement: Supplementary file 2 — Supplementary Material 2 [file 12879_2025_10566_MOESM2_ESM.docx]

Supplementary Table 2: Comparison of 17 risk variables between the internal validation cohort and the training cohort for the HBC prognostic model

| Factor | Internal validation group | Training group | P value |
| --- | --- | --- | --- |
| N | 121 | 121 |  |
| Complications |  |  | 1.00 |
| <3 | 64 (52.9%) | 65 (53.7%) |  |
| ≥3 | 57 (47.1%) | 56 (46.3%) |  |
| HCC |  |  | 1.00 |
| No | 65 (53.7%) | 65 (53.7%) |  |
| Yes | 56 (46.3%) | 56 (46.3%) |  |
| SV |  |  | 0.78 |
| No | 81 (66.9%) | 84 (69.4%) |  |
| Yes | 40 (33.1%) | 37 (30.6%) |  |
| PHF |  |  | 0.90 |
| No | 71 (58.7%) | 69 (57.0%) |  |
| Yes | 50 (41.3%) | 52 (43.0%) |  |
| HBVDNA |  |  | 0.60 |
| negative | 49 (40.5%) | 44 (36.4%) |  |
| positive | 72 (59.5%) | 77 (63.6%) |  |
| NLR, median (IQR) | 3.1 (1.8, 4.6) | 2.8 (1.8, 4.6) | 0.60 |
| PLR, median (IQR) | 71.8 (43.8, 101.4) | 71.8 (46.6, 101.4) | 0.87 |
| mono, median (IQR) | 0.3 (0.3, 0.6) | 0.3 (0.2, 0.5) | 0.35 |
| TB, median (IQR) | 23.7 (15.3, 41.6) | 24.0 (16.3, 37.3) | 0.80 |
| DB, median (IQR) | 10.5 (7.0, 22.2) | 10.5 (7.1, 17.5) | 1.00 |
| GGT, median (IQR) | 58.1 (29.6, 94.4) | 67.3 (31.7, 150.3) | 0.21 |
| ALP, median (IQR) | 95.0 (63.7, 138.0) | 99.9 (67.1, 140.0) | 0.69 |
| LDH, median (IQR) | 211.0 (162.0, 250.0) | 210.0 (163.0, 251.0) | 0.75 |
| AFU, median (IQR) | 18.0 (13.0, 24.0) | 19.0 (15.0, 25.0) | 0.33 |
| ADA, median (IQR) | 23.6 (17.4, 30.2) | 24.1 (17.8, 31.8) | 0.69 |
| Fib, median (IQR) | 1.6 (1.4, 2.2) | 1.7 (1.4, 2.3) | 0.30 |
| AFP, median (IQR) | 16.7 (3.8, 239.7) | 20.7 (4.3, 239.7) | 0.75 |
| Survival time, median (IQR) | 755.0 (220.0, 2067.0) | 1026.0 (203.0, 2026.0) | 0.60 |
| Survival status |  |  | 0.23 |
| alive | 51 (42.1%) | 41 (33.9%) |  |
| death | 70 (57.9%) | 80 (66.1%) |  |
