# Supplementary material for: Identification of multiple complications as independent risk factors associated with 1-, 3-, and 5-year mortality in hepatitis B-associated cirrhosis patients
Source: BMC Infect Dis. 2025 Feb 1;25:151. doi: 10.1186/s12879-025-10566-6 (PMC11786570; doi:10.1186/s12879-025-10566-6)
Supplement: Supplementary file 3 — Supplementary Material 3 [file 12879_2025_10566_MOESM3_ESM.docx]

Supplementary Table 1: Comparison of variables between the internal validation cohort and the training cohort for predicting multiple complications model

| Variables | Internal validation group | Training group | p-value |
| --- | --- | --- | --- |
| N | 121 | 121 |  |
| Complications |  |  | 1.00 |
| <3 | 64 (52.9%) | 65 (53.7%) |  |
| ≥3 | 57 (47.1%) | 56 (46.3%) |  |
| Gender |  |  | 1.00 |
| Woman | 29 (24.0%) | 29 (24.0%) |  |
| Male | 92 (76.0%) | 92 (76.0%) |  |
| HBVFH |  |  | 0.19 |
| No | 103 (85.1%) | 94 (77.7%) |  |
| Yes | 18 (14.9%) | 27 (22.3%) |  |
| EtOH |  |  | 1.00 |
| No | 109 (90.1%) | 110 (90.9%) |  |
| Yes | 12 (9.9%) | 11 (9.1%) |  |
| SH |  |  | 0.60 |
| No | 115 (95.0%) | 112 (92.6%) |  |
| Yes | 6 (5.0%) | 9 (7.4%) |  |
| HCC |  |  | 0.70 |
| No | 61 (50.4%) | 65 (53.7%) |  |
| Yes | 60 (49.6%) | 56 (46.3%) |  |
| HTN |  |  | 0.86 |
| No | 104 (86.0%) | 102 (84.3%) |  |
| Yes | 17 (14.0%) | 19 (15.7%) |  |
| DM |  |  | 0.33 |
| No | 94 (77.7%) | 101 (83.5%) |  |
| Yes | 27 (22.3%) | 20 (16.5%) |  |
| PE |  |  | 0.76 |
| No | 25 (20.7%) | 28 (23.1%) |  |
| Yes | 96 (79.3%) | 93 (76.9%) |  |
| SV |  |  | 0.78 |
| No | 81 (66.9%) | 84 (69.4%) |  |
| Yes | 40 (33.1%) | 37 (30.6%) |  |
| PHF |  |  | 1.00 |
| No | 68 (56.2%) | 69 (57.0%) |  |
| Yes | 53 (43.8%) | 52 (43.0%) |  |
| vomiting |  |  | 0.69 |
| No | 70 (57.9%) | 74 (61.2%) |  |
| Yes | 51 (42.1%) | 47 (38.8%) |  |
| AVT |  |  | 0.83 |
| No | 13 (10.7%) | 11 (9.1%) |  |
| Yes | 108 (89.3%) | 110 (90.9%) |  |
| HBVDNA |  |  | 1.00 |
| negative | 43 (35.5%) | 44 (36.4%) |  |
| positive | 78 (64.5%) | 77 (63.6%) |  |
| Age (years,median IQR) | 56.0 (48.0, 64.0) | 55.0 (45.0, 62.0) | 0.27 |
| BMI (median IQR) | 23.4 (21.5, 25.6) | 23.4 (21.1, 25.4) | 0.56 |
| WBC (10^9/L,median IQR) | 4.6 (2.8, 6.4) | 4.3 (2.9, 6.4) | 0.96 |
| NEU (10^9/L,median IQR) | 2.8 (1.6, 4.2) | 2.8 (1.6, 4.3) | 0.87 |
| NLR (median IQR) | 2.7 (1.8, 3.5) | 2.8 (1.8, 4.6) | 0.34 |
| HB (g/L,median IQR) | 121.0 (111.0, 137.0) | 121.0 (107.0, 134.0) | 0.45 |
| PLT (10^9/L,median IQR) | 70.0 (44.0, 106.0) | 67.0 (46.0, 100.0) | 0.64 |
| LYM (10^9/L,median IQR) | 1.0 (0.8, 1.5) | 1.0 (0.7, 1.3) | 0.33 |
| PLR (median IQR) | 62.9 (46.8, 97.5) | 71.8 (46.6, 101.4) | 0.41 |
| RBC (10^12/L,median IQR) | 3.7 (3.2, 4.2) | 3.7 (3.2, 4.2) | 0.99 |
| HCT (%,median IQR) | 35.4 (31.7, 40.7) | 35.4 (31.0, 39.3) | 0.45 |
| MONO (10^9/L,median IQR) | 0.3 (0.2, 0.5) | 0.3 (0.2, 0.5) | 0.87 |
| EOS (10^9/L,median IQR) | 0.1 (0.0, 0.1) | 0.1 (0.0, 0.1) | 0.27 |
| TB (μmol/L,median IQR) | 24.1 (17.6, 50.2) | 24.0 (16.3, 37.3) | 0.62 |
| DB (μmol/L,median IQR) | 10.7 (7.4, 21.1) | 10.5 (7.1, 17.5) | 0.74 |
| ALT (U/L,median IQR) | 42.1 (26.2, 77.2) | 40.6 (26.2, 66.1) | 0.52 |
| AST (U/L,median IQR) | 57.1 (32.9, 94.0) | 47.8 (30.4, 84.1) | 0.38 |
| ALB (g/L,median IQR) | 33.0 (27.9, 37.5) | 32.8 (28.9, 37.6) | 0.95 |
| GLO (g/L,median IQR) | 33.0 (28.8, 37.7) | 31.5 (26.5, 35.9) | 0.10 |
| GGT (U/L,median IQR) | 71.8 (35.8, 174.5) | 67.3 (31.7, 150.3) | 0.35 |
| CHE (U/L,median IQR) | 3507.0 (2466.0, 4749.0) | 3507.0 (2242.0, 4896.0) | 1.00 |
| ALP (U/L,median IQR) | 109.2 (67.1, 144.3) | 99.9 (67.1, 140.0) | 0.52 |
| LDH (U/L,median IQR) | 224.0 (175.0, 268.0) | 210.0 (163.0, 251.0) | 0.23 |
| TBA (μmol/L,median IQR) | 24.0 (9.5, 53.7) | 22.7 (9.2, 53.7) | 0.89 |
| Lactate (mmol/L,median IQR) | 2.7 (2.2, 3.0) | 2.7 (2.2, 3.0) | 0.46 |
| RBP (mg/L,median IQR) | 18.6 (13.5, 27.4) | 19.5 (13.3, 28.5) | 0.79 |
| AFU (U/L,median IQR) | 19.0 (15.0, 25.0) | 19.0 (15.0, 25.0) | 0.98 |
| MAO (U/L,median IQR) | 5.2 (3.8, 6.6) | 4.9 (3.5, 6.5) | 0.39 |
| ADA (U/L,median IQR) | 25.4 (19.4, 32.8) | 24.1 (17.8, 31.8) | 0.35 |
| B2M (mg/ml,median IQR) | 2.8 (2.1, 4.0) | 2.9 (2.2, 4.0) | 0.88 |
| serum potassium (mmol/L,median IQR) | 3.8 (3.5, 4.3) | 3.8 (3.6, 4.2) | 0.74 |
| serum sodium (mmol/L,median IQR) | 140.3 (138.1, 142.3) | 140.1 (137.3, 142.5) | 0.67 |
| serum calcium (mmol/L,median IQR) | 2.0 (1.9, 2.2) | 2.0 (1.9, 2.2) | 0.99 |
| serum ferritin (umol/L,median IQR) | 19.1 (11.0, 30.0) | 19.1 (9.9, 28.7) | 0.63 |
| serum phosphate (mmol/L,median IQR) | 1.0 (0.8, 1.1) | 1.0 (0.9, 1.1) | 0.17 |
| BUN (mmol/L,median IQR) | 5.6 (4.6, 6.8) | 5.6 (4.7, 7.4) | 0.58 |
| Cr (μmol/L,median IQR) | 67.0 (55.3, 78.0) | 69.9 (58.0, 79.0) | 0.45 |
| PT (second,median IQR) | 14.9 (13.7, 16.7) | 14.8 (13.7, 16.8) | 0.97 |
| APTT (second,median IQR) | 36.2 (30.2, 43.5) | 36.5 (30.9, 42.7) | 0.63 |
| TT (second,median IQR) | 21.1 (19.7, 24.0) | 21.1 (19.7, 23.2) | 0.57 |
| Fib (g/L,median IQR) | 1.8 (1.5, 2.5) | 1.7 (1.4, 2.3) | 0.63 |
| AT3 (mg/dl,median IQR) | 71.9 (54.1, 84.4) | 73.2 (53.4, 85.3) | 0.78 |
| PTA (%,median IQR) | 58.7 (50.8, 68.7) | 59.4 (50.8, 69.6) | 0.99 |
| AFP (ng/ml,median IQR) | 19.6 (7.3, 239.7) | 20.7 (4.3, 239.7) | 0.80 |

**Note.** ADA, adenosine deaminase; AFP, alpha-fetoprotein; AFU, alpha-l-fucosidase; ALB, albumin; ALP, alkaline phosphatase; ALT, alanine aminotransferase; APTT, activated partial prothrombin time; AST, aspartate aminotransferase; AT3, antithrombin Ⅲ; AVT, Antiviral Therapy; B2M, Beta-2-microglobulin; BMI, Body Mass Index; BUN, blood urea nitrogen; CHE, cholinesterase; Cr, creatinine; DB, direct bilirubin; DM, Diabetes Mellitus; EOS, eosinophil; EtOH, Ethanol/Alcohol History; Fib, fibrinogen; GGT, γ-glutamyl transpeptadase; GLO, globulin; HB, hemoglobin; HBVFH, Family History of Hepatitis B Virus; HCC, Hepatocellular Carcinoma; HCT, hematocrit; HTN, Hypertension; IQR, interquartile range; LA, lactic acid; LDH, lactic dehydrogenase; LYM, lymphocyte; MAO, monoamine oxidase; MONO, monocyte; NEU, neutrophil; NLR, neutrophil to lymphocyte ratio; PE, Palmar Erythema; PHF, Post-hospitalisation fever; PLR, platelet to lymphocyte ratio; PLT, platelet; PT, Prothrombin time; PTA, prothrombin time activity; RBC, red blood cell; RBP, retinol binding protein; SH, Smoking History; SV, Spider Nevi; TB, total bilirubin; TBA, total bile acid; TBIL, total bilirubin; TT, thrombin time; WBC, white blood cell. *P < 0.05. Categorical variables were analyzed using Fisher's exact test, and continuous variables were evaluated with the Wilcoxon rank-sum test to calculate P-values.
